# Revascularisation difficulties in acute cardiac syndrome as debut of Takayasu arteritis

**DOI:** 10.1007/s12471-020-01523-w

**Published:** 2020-11-30

**Authors:** A. Riaño Ondiviela, J. Alameda Serrano, A. Lukic Otanovic, J. R. Ruiz Arroyo

**Affiliations:** Cardiology Department, Hospital Clínico Universitario Lozano Blesa, Zaragoza, Spain

**Fig. 1:**
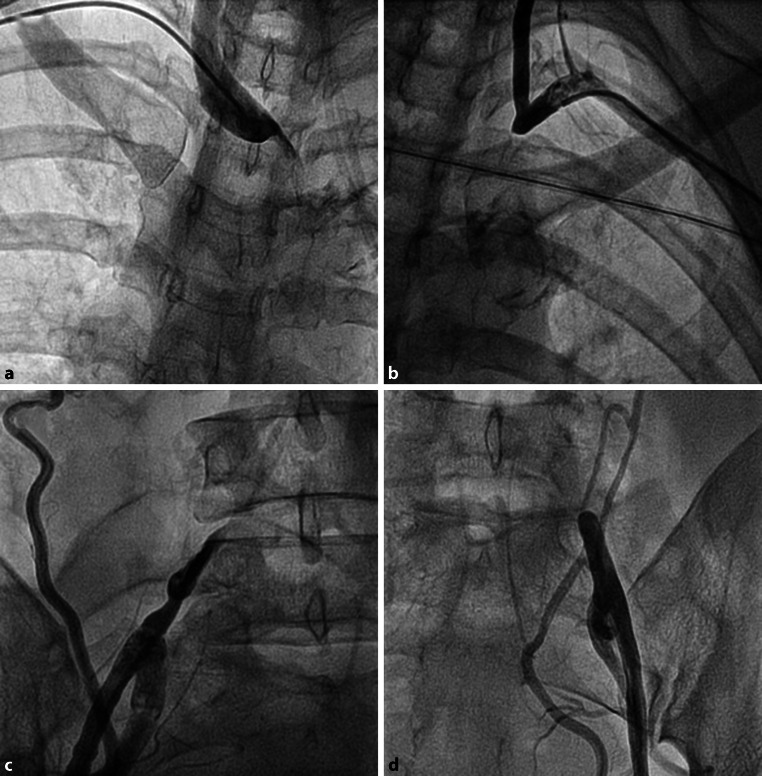
Angiography showing occluded vascular access sites. **a** Right brachiocephalic artery. **b** Aberrant left brachiocephalic artery. **c** Right and **d** left common femoral artery

A 44-year-old woman without cardiovascular risk factors presented with sudden oppressive chest pain. There were heart failure signs and a right carotid murmur; the left radial and brachial pulse were absent. Electrocardiography showed a 6-mm ST-segment elevation in V2–V6, suggesting anterior myocardial infarction. She was referred for emergent percutaneous coronary revascularisation and several insertion sites were tried to gain vascular access, but without success (Fig. 1). Percutaneous recanalisation and stent implantation in the right brachiocephalic branch were necessary to perform coronary angiography and angioplasty with drug-eluting stent implantation in a critical stenosis of the proximal part of the left descending coronary artery.

In this case, the clinical presentation, physical examination and angiographic findings suggested large-vessel vasculitis due to Takayasu disease [1–3]. Takayasu arteritis must be included in the differential diagnosis of acute coronary syndrome, especially in young women without cardiovascular risk factors [4].
